# Functional connectivity of default mode network in non-hospitalized patients with post-COVID cognitive complaints

**DOI:** 10.3389/fnins.2025.1576393

**Published:** 2025-04-10

**Authors:** Derek Madden, Tressie M. Stephens, Jim Scott, Christen O’Neal Swann, Kiana Prather, Jordan Hoffmeister, Lei Ding, Ian F. Dunn, Andrew K. Conner, Han Yuan

**Affiliations:** ^1^Stephenson School of Biomedical Engineering, Gallogly College of Engineering, The University of Oklahoma, Norman, OK, United States; ^2^Department of Neurosurgery, The University of Oklahoma Health Sciences Center, Oklahoma City, OK, United States; ^3^Department of Psychiatry and Behavioral Sciences, The University of Oklahoma Health Sciences Center, Oklahoma City, OK, United States; ^4^Institute for Biomedical Engineering, Science, and Technology, University of Oklahoma, Norman, OK, United States

**Keywords:** COVID-19, functional magnetic resonance imaging, resting state functional connectivity, default mode network, cognitive impairment, posterior cingulate cortex

## Abstract

**Introduction:**

Neurologic impairment is common in patients with acute respiratory syndrome coronavirus-2 (SARS-CoV-2) infection. While patients with severe COVID have a higher prevalence of neurologic symptoms, as many as one in five patients with mild COVID may also be affected, exhibiting impaired memory as well as other cognitive dysfunctions.

**Methods:**

To characterize the effect of COVID on the brain, the current study recruited a group of adults with post-COVID cognitive complaints but with mild, non-hospitalized cases. They were then evaluated through formal neuropsychological testing and underwent functional MRI of the brain. The participants in our study performed nearly as expected for cognitively intact individuals. Additionally, we characterized the functional connectivity of the default mode network (DMN), which is known for cognitive functions including memory as well as the attention functions involved in normal aging and degenerative diseases.

**Results:**

Along with the retention of functional connectivity in the DMN, our results found the DMN to be associated with neurocognitive performance through region-of-interest and whole-brain analyses. The connectivity between key nodes of the DMN was positively correlated with cognitive scores (*r* = 0.51, *p* = 0.02), with higher performers exhibiting higher DMN connectivity.

**Discussion:**

Our findings provide neuroimaging evidence of the functional connectivity of brain networks among individuals experiencing cognitive deficits beyond the recovery of mild COVID. These imaging outcomes indicate expected functional trends in the brain, furthering understanding and guidance of the DMN and neurocognitive deficits in patients recovering from COVID.

## 1 Introduction

Since the start of the COVID-19 pandemic, a constellation of COVID-related symptoms involving various organ systems have been reported. Although COVID was well-known for causing respiratory complications, its effect on the central nervous system was not established until months into the pandemic (Mao et al., 2020). One of the more common Post-Acute Sequela of SARS-CoV-2 infection (PASC; Thaweethai et al., 2023)—cognitive impairment referred to as “COVID brain fog”—affects as many as 27% of the patients suffering from PASC (Rivera-Izquierdo et al., 2022). PASC can affect wide ranges of patients including both severe and mild COVID patients through memory loss, reduced processing speed, deficits in language and executive function, and lapses in attention (Hampshire et al., 2021; Venkataramani and Winkler, 2022). A recent study reported that 36.4% of patients hospitalized with COVID had neurologic symptoms, while the prevalence was 45.5% in those with severe COVID (Mao et al., 2020). Up to 21% of patients with mild COVID may also exhibit neurologic symptoms such as memory impairment (Del Brutto et al., 2021). Although the majority of the individuals eventually recover or improve from their cognitive impairment (Coronavirus and the Nervous System, 2023), it is unclear whether COVID may pose an increased risk for cognitive impairment later in life or early-onset dementia (Miners et al., 2020).

Inflammatory changes and hypoxic brain injury have been proposed as potential mechanisms of neurologic dysfunction in COVID patients, both of which have been described in the pathophysiology of Alzheimer’s Disease (AD; Iadecola et al., 2020; Jha et al., 2021). Structural MRI imaging in patients with acute or subacute COVID have been associated with diffuse white matter hyperintensities, commonly affecting the periventricular regions, usually reported as “chronic white matter changes” or “chronic microvascular ischemia” which is a finding often seen in older individuals with concomitant vascular risk factors such as hypertension, diabetes, and hyperlipidemia and can be associated with vascular dementia (Egbert et al., 2020; Lu et al., 2020). Such structural findings indicate a mechanism of abnormal cognitive neural circuits underlying the COVID cognitive impairment. Preliminary studies in mice have shown similar white matter changes, with increased white-matter-selective microglial reactivity resulting in neural inflammation (Fernández-Castañeda et al., 2022). Nonetheless, the relevance of these structural abnormalities in cognitive dysfunction is not fully established.

Functional Magnetic Resonance Imaging (fMRI) has become a widespread approach in studying brain function among the cognitively impaired and neurologically intact people. Resting state fMRI originally revealed the default mode network (DMN), which is recognized for characterizing the trajectory of normal aging as well as degenerative progression in AD (Buckner et al., 2005; Fox et al., 2005; Greicius et al., 2004; Raichle et al., 2001). The DMN is composed of several regions known to be associated with various cognitive functioning (Buckner et al., 2008; Mevel et al., 2011): The posterior cingulate cortex (PCC) is associated with episodic memory encoding (Natu et al., 2019); the medial prefrontal cortex (mPFC) is linked with social cognition (Amodio and Frith, 2006); the medial temporal lobe is reported to contribute to episodic memory and episodic future thinking (Race et al., 2011); and the parietal cortex is associated with attention function (Behrmann et al., 2004). Decrease of the functional connectivity within the DMN has been recognized in advanced aging compared to young adults (Andrews-Hanna et al., 2007; Damoiseaux et al., 2008), as well as in AD than healthy controls (Greicius et al., 2004; Sorg et al., 2007). However, the progressive trajectory of the connectivity is not monotonic, especially across the middle-age to elderly range (Andrews-Hanna et al., 2007; Ball et al., 2017; Chiesa et al., 2019; Staffaroni et al., 2018; Zonneveld et al., 2019; Chen et al., 2022). A positive association such that higher retained functions is aligned with greater connectivity in the DMN whereas more decline accompanying less connectivity has been observed in the elderly normal (Andrews-Hanna et al., 2007). Importantly, brain connectivity might also be seen in a negative association with cognitive functionality: especially, elevated connectivity has been seen in certain cases of cognitive decline prior to any development of dementia but later being converted to dementia (Sorg et al., 2007; Damoiseaux et al., 2012; De Vogelaere et al., 2012; Gardini et al., 2015; Chiesa et al., 2019), possibly via a compensatory mechanism (Amieva et al., 2014; Kanishka Jha, 2023).

Despite structural neuroimaging findings reported in COVID patients, studies examining the brain networks that underlie the memory and other cognitive functions are limited (Fischer et al., 2020; Esposito et al., 2022; Zhang et al., 2022; Díez-Cirarda et al., 2023; Muccioli et al., 2023; Fineschi et al., 2024). Especially, most of the existing reports on the functional connectivity in post-COVID patients included cases with severity that ranges from mild, moderate to severe, despite that recent studies have indicated that hospitalized cases present distinct functional networks from non-hospitalized cases (Díez-Cirarda et al., 2023). It was not clear if the cognitive impairment in post-COVID patients, especially in a group of homogenous severity, would be in any way related to the neural circuits underlying those cognitive functions, and if they were, whether the brain-function association would be in a positive or negative manner that might suggest completely different mechanisms. The aim of this study was thus to examine a group of COVID patients with subjective cognitive impairment following COVID infection using resting state fMRI (rs-fMRI) in conjunction with neuropsychological evaluation. We characterized the functional connectivity of the DMN using a seed-based approach that has been consistently utilized before and produced benchmark values in healthy and AD populations (Andrews-Hanna et al., 2007). Furthermore, we investigated the association between the DMN connectivity and cognitive performance across the studied individuals. These imaging outcomes would guide subsequent efforts to characterize the trajectory of the impairment and explore treatment options.

## 2 Materials and methods

### 2.1 Participants

The study protocol was approved by the Institutional Review Board at the University of Oklahoma Health Sciences Center. Subjects from the Oklahoma City area with a history of positive SARS-CoV-2 polymerase chain reaction (PCR) and/or antigen rapid test in the previous 18-month period along with self-reported cognitive impairment were recruited. Each participant provided informed, written consent to participate. Considering that anxiety and depression have been shown to impact the DMN (Drevets et al., 2008; Greicius et al., 2007; Sheline et al., 2010), we excluded subjects with scores of >21 on the Beck Anxiety Index (BAI) or > 19 on the Beck Depression Index (BDI). Other exclusion criteria included pre-existing neurological or psychiatric disorders, illicit drug use, and use of medications that may affect neural activity.

### 2.2 Neuropsychological measures

Participants completed a battery of neuropsychological tests for evaluation of cognitive function, including the Repeatable Battery for the Assessment of Neuropsychological Status (RBANS) and the Montreal Cognitive Assessment (MoCA). The RBANS is a clinically validated, repeatable neuropsychological examination often used to evaluate neurological status of individuals suffering from neurologic disease or head injury (Randolph et al., 1998), which provides information for a variety of functions by dividing performance into five different indices: Immediate Memory, Visuo-construction, Language, Attention, and Delayed Memory. The MoCA is another neuropsychological examination that has been proficient in diagnosing and differentiating cognitive impairment (Benge and Kiselica, 2021). As the MoCA score has been established as a screening tool to detect mild cognitive impairment (Nasreddine et al., 2005), we used the RBANS total score as the primary scale for neurocognitive evaluation. We reported RBANS standard scores that are normative scores based on the individuals’ gender and age according to the scoring manual (Randolph et al., 1998). We also stratified our participants into two subgroups of lower and higher performers based on their median scores on the RBANS total.

### 2.3 MRI acquisition

All imaging was performed on a GE Signa 3 T Architect scanner (GE Healthcare, Milwaukee, Wisconsin) at the OU Medical Center Campus. A brain-dedicated receive-only 16-channel coil array was used for MRI signal reception. A T2*-sensitive gradient echo spiral in/out pulse sequence was used to acquire resting state functional MRI. The following acquisition parameters were used for fMRI: field of view/slice/gap = 240/4.0/1.0 mm, axial slices per volume = 41, acquisition matrix = 64 × 64, voxel size = 3.750 × 3.750 × 4.000 mm^3^, repetition/echo time = 2500/30 ms, acceleration factor R = 2 in the phase encoding (anterior–posterior) direction, flip angle = 90°, sampling bandwidth = 250 kHz, number of volumes = 239. Additionally, a spoiled gradient-recalled sequence for T1 contrast was used to acquire structural MRI, with the following parameters: FOV = 240 mm, axial slices per slab = 176, slice thickness = 1.0 mm, image matrix = 256 × 256, TR/TE = 7.7/3.1 ms, acceleration factor R = 2, flip angle = 8°, inversion time TI = 800 ms, sampling band-width = 31.2 kHz.

### 2.4 Data preprocessing

The preprocessing of resting state fMRI data was performed using Analysis of Functional Neuroimages (AFNI) per previously published protocol (Yuan et al., 2014; Yuan et al., 2016; Yuan et al., 2017; Zhang et al., 2023). The first five volumes of each run were excluded from analysis to allow the blood-oxygen-level-dependent (BOLD) signal to reach steady state. Major processing steps included slice timing and rigid-body motion correction, spatial smoothing with a Gaussian kernel (FWHM = 6 mm), and temporal filtering with a bandpass filter (0.005–0.1 Hz). In addition, six affine motion parameters, signal from a ventricular region of interest, and signal from a region centered in the white matter were regressed out from the dataset. Data points of excessive motion (root mean square larger than 0.3 mm) were excluded from regression and correlation analysis using the *censoring* option implemented in AFNI (afni_proc.py). Specifically, the L2-norm of motion parameters estimated from motion registration was calculated per run and those time points of amplitude larger than 0.3 mm were censored/excluded in the regression or in the later calculation of connectivity. Notably the band-pass filtering was implemented in a linear regression fashion. Therefore, censoring out points of extraordinary motion did not lead to unusual edge effects as seen in filtering using a convolution form. The whole-brain global signal was *not* removed as this may lead to spurious anti-correlation (Fox et al., 2009; Murphy et al., 2009). The fMRI data of each participant was first spatially co-registered to high-resolution anatomical images and then to the Talairach and Tournoux template brain (Talairach and Tournoux, 1988) for spatial normalization.

### 2.5 Resting state functional connectivity analysis

Resting state functional connectivity was computed as the Pearson’s correlation in regard to a seed region (Biswal et al., 1995). For this study, the correlation associated with the PCC was of interest due to its integration into the DMN. The seed was thus defined as a 5-mm-radius sphere centered at the PCC (−1, −50, 26) in the Talairach space, according to the coordinates previously established in an independent study (Andrews-Hanna et al., 2007). To examine specific regions within the DMN, additional regions of interest (ROIs) were defined in accordance with Andrews-Hanna et al. (2007) representing the mPFC (1, 40, 16), the lateral parietal cortex (LatPar; L: −45, −67, 26; R: 53, −65, 26), the hippocampal formations (HF; L: −23, −25, −12; R: 23, −25, −12), and the parahippocampal cortex (PHC; L: −25, −39, −10; R: 25, −39, −10). Spherical masks were generated which encompassed these ROIs and the voxel connectivity values within each ROI were extracted. These connectivity values were then averaged to compute the average connectivity of each region with the PCC. Through the masking process, the connectivity values were summed across all ROIs to determine the overall DMN connectivity while also retaining average connectivity values for each ROI. These regions were further investigated as individual seeds, where their connectivity values with the remaining ROIs were determined using the same methods as above.

Correlation values representing the functional connectivity were produced by extracting the pre-processed BOLD time course from a seed region, averaging the signals within the seed region, then computing the correlation coefficient between the seed time course and the time course from another region of interest different from the seed region (namely the target region), or from all other brain voxels. The resulted correlation coefficients were converted to a normal distribution using the Fisher’s z transform. This transform resulted in voxel z-score maps for individuals that were further subject to group-level analysis. In reporting our results, we listed r values for direct comparison with the previous studies on healthy aging normal (Andrews-Hanna et al., 2007). All statistical evaluations on the connectivity values, however, were based on the transformed z values.

In addition to the ROI analysis, whole-brain connectivity with regards to the PCC as the seed was also evaluated using 3dttest++ on individuals’ connectivity maps, assigning a t-score and *p*-value for each voxel to generate an averaged connectivity map of z values. Furthermore, connectivity maps were stratified based on performance levels of cognitive tests according to the RBANS total scores. Participants were split into two subgroups - the higher scorers and the lower scorers. The PCC-seeded connectivity map for each subgroup was then generated once again by averaging the time series of PCC-masked voxels and generating the connectivity value of each voxel in the brain using the ordinary least squares regression on each voxel’s time series. With the connectivity maps for each participant, the *t*-tests were used for generation of the mean connectivity value and t value for each voxel within groups. All connectivity maps were thresholded at *p*_corrected_ < 0.05 determined using the AFNI program 3dClustSim (Yuan et al., 2014).

### 2.6 Brain-age association

In order to examine the aging effect on the connectivity values and also to benchmark with the prior studies that examined the aging trajectory of connectivity, we analyzed the covariation between functional connectivity of DMN through the PCC-mPFC connectivity and the age of years among all subjects, while controlling the years of education as a confounding factor. In addition, the individual values were also stratified based on the cognitive performance. The connectivity values in higher and lower performers were separately compared with their ages.

### 2.7 Brain-function association

To examine the functional relevance of the neuroimaging outcomes, we analyzed the covariation between the functional connectivity of the DMN and the cognitive performance indexed by the RBANS total scores. Importantly, the covariations were calculated with connectivity controlled for age and gender. Because the RBANS total scores were already age- and gender-corrected, the age/gender correction was performed on the connectivity values only. Specifically, in MATLAB R2021b, linear regression models relating connectivity to age and gender were generated. The connectivity residuals were then calculated, and these residuals were correlated with RBANS total performance. Additionally, using the *partialcorr* command in MATLAB, respective r and *p* values were calculated, while controlling the years of education as a confounding factor.

Furthermore, at the whole brain level we explored the regions where the PCC-seeded functional connectivity showed covariation with the cognitive performance indexed by the RBANS total scores. Only voxels revealing significant connectivity in conjunction with significant covariation with cognitive performance were imaged. In particular, the covariation between PCC-seeded connectivity matrix produced for all patients and their corresponding RBANS total scores was evaluated to produce a statistic map, which was masked by a map of significant connectivity regarding the PCC seed. Regions were then identified by applying a threshold of *p*_corrected_ < 0.05 determined using the AFNI program 3dClustSim (Yuan et al., 2014). For any identified clusters, the average regional connectivity with the PCC was calculated by averaging the connectivity z scores of each voxel within the ROI. Then, those extracted ROI values were compared to the RBANS total scores across subjects with age and gender controlled, as was done with the DMN.

### 2.8 Statistical analysis

When evaluating the difference between two subgroups, a two-sample *t* test was applied if the normality was met. To test the normality of our data, we employed a corrected Kolmogorov–Smirnov test or LF KS test (Lilliefors, 1967; Öner and Deveci Kocakoç, 2017), with assumptions of unknown population mean and variance. When normality was not met, the Mann–Whitney U test was used instead. When there was a significant difference, the effect size was calculated as the Cohen’s d, which is defined as the mean difference between two groups divided by the pooled standard deviation.

## 3 Results

### 3.1 Demographic and clinic data

A total of 24 participants who met the inclusion/exclusion criteria were recruited and completed the study protocol from October 2021 to December 2022. Three participants’ fMRI volumes were discarded due to incomplete coverage of the brain and/or time series, resulting in a sample of 21 participants to be included in the final analysis, because our main hypotheses concern both the neurocognitive evaluates and neuroimaging analysis. The demographics and clinical characteristics are listed in Table 1. All subjects presented with cognitive complaints at least 1 month after a positive COVID test. This cohort of participants are largely middle-aged adults (37.33 ± 10.08 years, range 26 to 60 years) and all completed high-school education (15.38 ± 1.07 years, range 13 to 16 years). There were 16 female and 5 male subjects. All subjects were considered as mild COVID patients: no subjects were admitted to intensive care unit (ICU) or hospitalized. At their MRI scans, the average time since COVID contraction was 9.45 ± 5.96 months. All participants had a BDI score lower than 19 (6.57 ± 5.08) and a BAI score lower than 21 (4.10 ± 3.94), which is consistent with the inclusion/exclusion criteria.

**Table 1:** Demographic and clinical characteristics.

Group	All subjects (*n* = 21)	Higher performers (*n* = 11)	Lower performers (*n* = 10)
Years of age	37.33 (10.09)	37.82 (9.30)	36.80 (11.37)
Years of education	15.38 (1.07)	15.91 (0.30)	14.80 (1.32)^*^
Months since diagnosis	9.45 (5.96)	9.82 (5.24)	9.05 (6.64)
Beck depression index	6.57 (5.08)	6.91 (6.16)	6.20 (3.85)
Beck anxiety index	4.10 (3.94)	3.55 (3.86)	4.70 (4.14)
Repeatable battery for the assessment of neurological status			
Total	99.53 (12.06)	107.55 (9.94)	91.10 (6.67)^#^
Immediate memory	103.11 (13.05)	108.45 (9.96)	98.30 (13.86)
Visuo-construction	96.95 (17.77)	104.73 (18.74)	91.10 (13.95)
Language	99.16 (10.23)	102.55 (9.45)	95.10 (8.89)
Attention	100.68 (14.97)	109.73 (11.78)	90.90 (10.01)*^Δ^*
Delayed memory	99.26 (10.40)	102.55 (12.07)	93.80 (6.71)
Montreal cognitive assessment			
Total	26.47 (1.81)	26.82 (1.47)	25.90 (2.28)
Visuo-spatial	4.38 (0.80)	4.27 (0.90)	4.50 (0.71)
Naming	3.00 (0.00)	3.00 (0.00)	3.00 (0.00)
Attention	5.57 (0.87)	5.91 (0.30)	5.20 (1.14)
Language	2.52 (0.81)	2.81 (0.40)	2.20 (1.03)
Abstraction	2.00 (0.00)	2.00 (0.00)	2.00 (0.00)
Delayed memory	2.95 (1.24)	2.91 (1.45)	3.00 (1.05)
Orientation	5.95 (0.22)	5.91 (0.30)	6.00 (0.00)

Numbers in () are standard deviation. *indicates significant difference at *p* = 0.01 with Cohen’s *d* = 1.14. #indicates significant difference at *p* = 0.0003 with Cohen’s *d* = 1.85, Δindicates significant difference at *p* = 0.001 with Cohen’s *d* = 1.65.

In terms of overall cognitive function, the participants exhibited MoCA scores of 26.47 ± 1.81 (range 22 to 29), which is above the cutoff of 26 for mild cognitive impairment (Nasreddine et al., 2005). In the evaluation of specific cognitive functions, this cohort of participants have scored RBANS totals of 99.71 ± 11.85 (range: 82 to 134). Compared to normal controls provided in the scoring manual (Randolph et al., 1998), the participants performed at nearly the expected level of 100 on average, which is in the normal range according to the database of healthy normals provided in the RBANS scoring manual. We then separated participants based on RBANS performance into the higher (11 participants) and lower (10 participants) performers according to whether their RBANS total scores were higher or lower than the median score of 101. Then we evaluated the two subgroups in their demographic and clinical characteristics, as shown in Table 1. All metrics except the age of years and the MOCA did meet the normality assumption. The two subgroups differed significantly in their RBANS total scores (*p* < 0.01, Cohen’s *d* = 1.85). The lower performers scored 91.10 ± 6.67 (range: 82 to 100) and the higher performers scored 107.55 ± 9.94 (ranged from 101 to 134). Three out of the 10 lower performing participants scored at 85 or lower on the RBANS total, which are considered lower than normative scores. Still, this subgroup represents three out of 21 participants scoring below 85, a frequency that is consistent with what would be expected in the normal population in this age range. Furthermore, the higher and lower performers did not differ in their ages (*p* = 0.38), or the length of time since diagnosis (*p* = 0.43). The only other significant difference between the higher and lower performers was years of education, with the higher scorers generally further educated at 15.91 years on average compared to 14.80 years among the lower scorers (*p* = 0.01, Cohen’s *d* = 1.14). This trend is expected, as RBANS performance has been shown to be positively correlated with education in previous studies (Collinson et al., 2014), and the scores are normalized for age but not education. Importantly, the differences in BAI and BDI scores between higher and lower performers were not significant. None of the MoCA total scores or any subdomain MoCA scores differed between higher and lower performers. Because these COVID subjects of our study did not differ from normative scores, yet they presented varying level of cognitive performance and differed between the higher and lower performers, our following report of the neuroimaging data focused on the results in association with the cognitive performance while we also explored the connectivity patterns within each subgroups.

In terms of other neurological symptoms, among all participants, 95.24% reported headaches, 85.71% reported muscle aches (myalgia), 76.19% reported brain or memory fog, 57.14% reported decreased or loss of small, 28.57% reported decrease/loss of taste. Chi-squared (χ^2^) tests were used to evaluate whether the two subgroups of higher and lower cognitive performers differed in any way when reporting these neurological symptoms. Results found that none of the symptoms were significantly different between the higher vs. lower cognitive performers (*p* = 0.23 for headaches, *p* = 0.32 for myalgia, *p* = 0.34 for brain or memory fog, *p* = 0.25 for decreased or loss of small, *p* = 0.14 for decrease/loss of taste).

### 3.2 Resting state functional connectivity of default mode network

**Figure 1:**
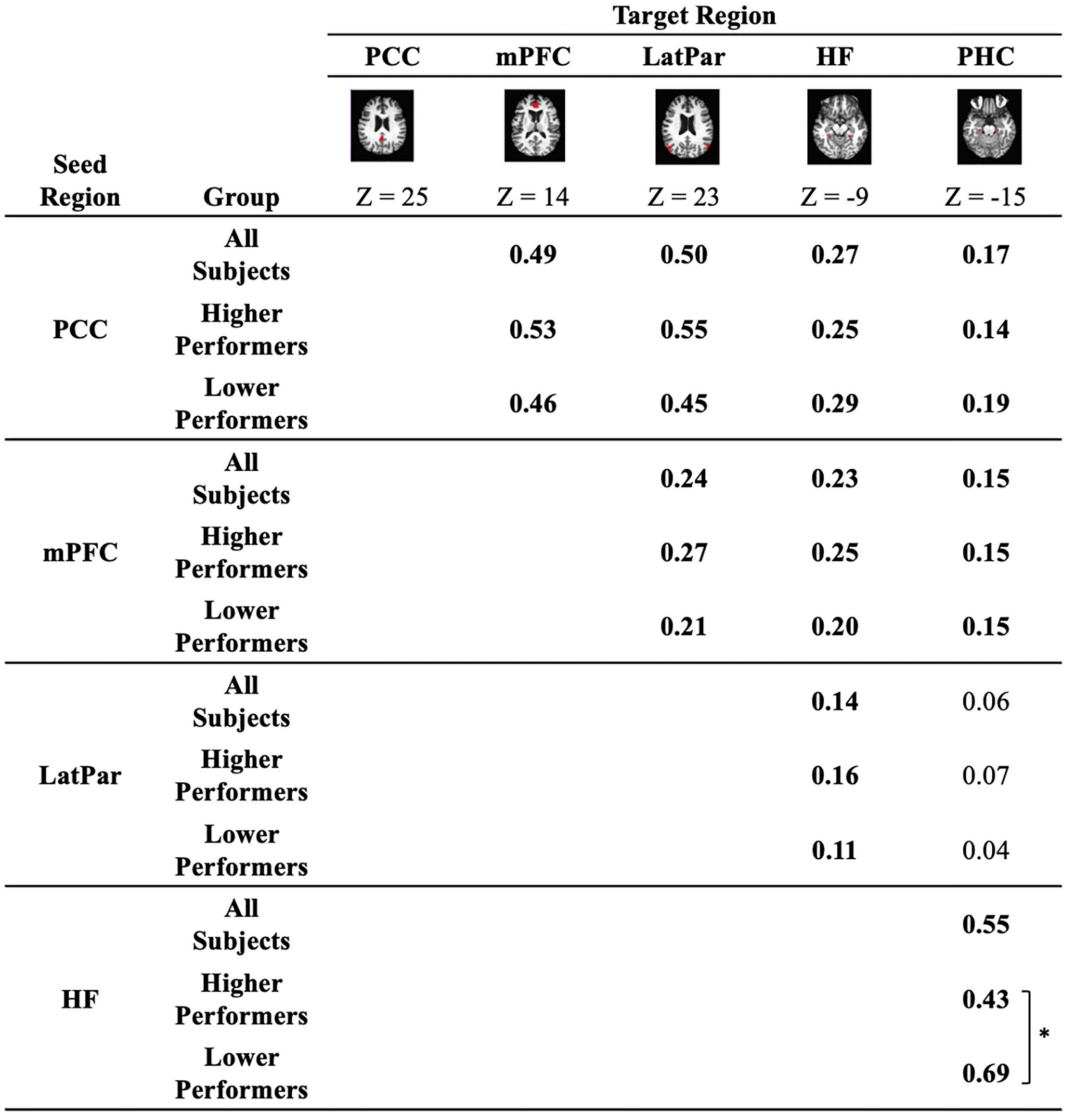
Functional connectivity between key regions of the default mode network. Correlation coefficients between *a priori* seed and target regions that comprise the default network are quantified in all participants as well as in the sub-groups of lower performers and higher performers on the repeatable battery for the assessment of neuropsychological status (RBANS) total scores. The regions illustrated in red are *a priori* regions of interest, including the medial prefrontal cortex (mPFC), the posterior cingulate cortex (PCC), the bilateral lateral parietal cortex (LatPar), the bilateral hippocampal formation (HF), and the bilateral parahippocampal cortex (PHC). Connectivity values significantly different from zero are highlighted in bold (*p* < 0.05). * indicates difference between subgroups of lower and higher performers (*p* < 0.05).

To ensure that regional measurements were made in an unbiased manner, all regions of interest (ROIs) used in the present analyses were defined according to a prior report in an independent dataset (Andrews-Hanna et al., 2007). The key regions of DMN are illustrated in Figure 1. We first examined the functional correlations between mPFC and PCC regions, which represent two major components of the DMN and are recognized for being compromised in aging. All connectivity values met the normality assumption. Our results found that the DMN exhibited an overall PCC-mPFC connectivity value of 0.49. Other DMN nodes with significant functional connectivity included the PCC-LatPar with connectivity value of 0.50, and the HF-PHC with connectivity of 0.55. Overall, the DMN nodes exhibited an averaged connectivity of 0.45. Furthermore, we stratified the ROI connectivity values based on the participants’ cognitive performance, as shown in Figure 1. Between the higher and lower performers, the connectivity values exhibited marginal differences with the overall trend of higher connectivity values observed in higher performer among the PCC, the mPFC and the bilateral parietal lobules. However, a reversed exception is noted in the connectivity between the hippocampal formation and the parahippocampal cortex in that higher connectivity is present in the lower performers compared to the higher performers (*p* < 0.05, Cohen’s *d* = 0.76).

### 3.3 Whole-brain functional connectivity of default mode network

**Figure 2:**
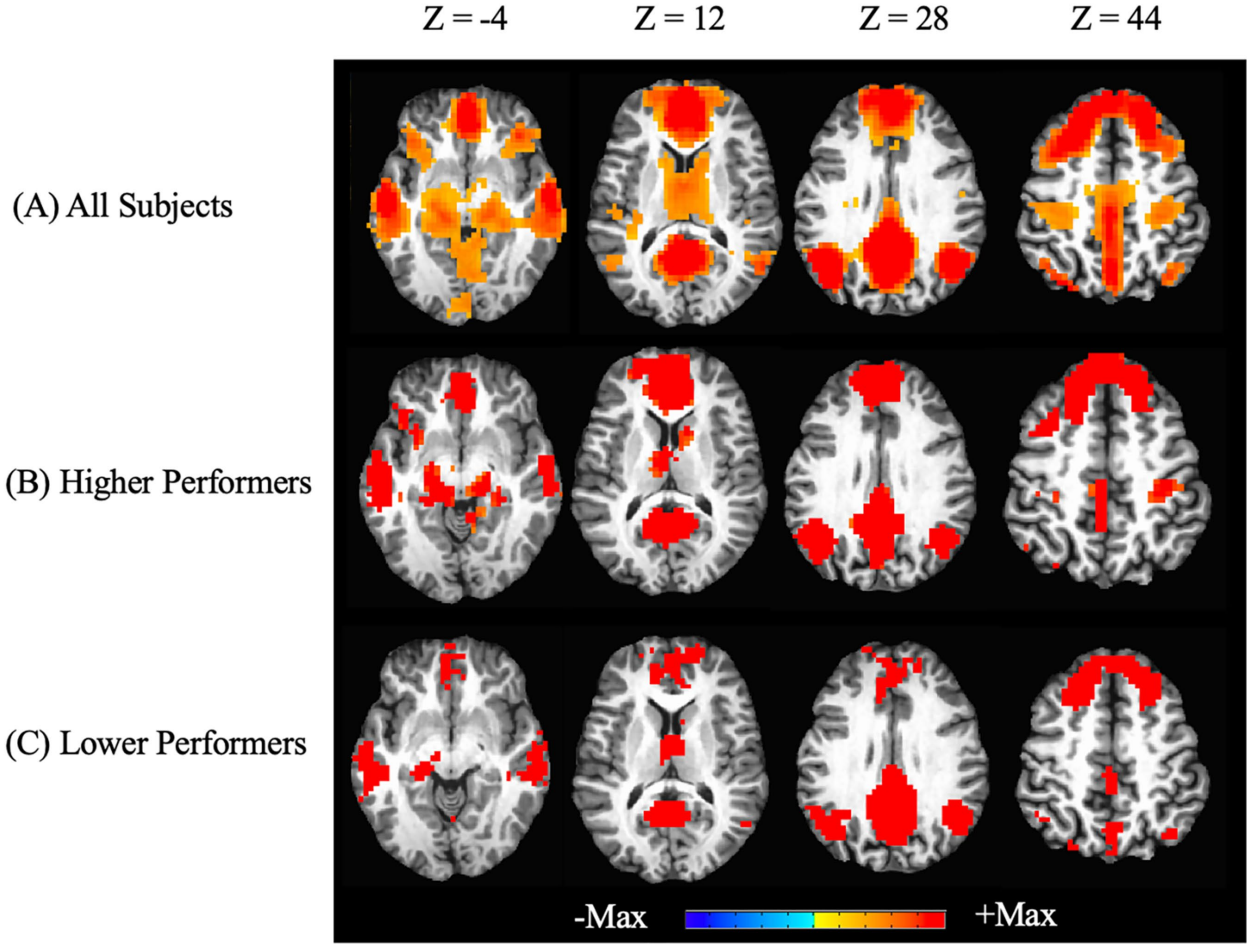
Whole-brain resting state functional connectivity of the default mode network. Functional connectivity seeded from the posterior cingulate cortex are graphically overlaid on an individual participant’s MRI (*p*_corrected_ < 0.05). Images at different Z positions in the Talaraich coordinates are shown. Positive correlations with the PCC as the seed are shown in all participants **(A)**, as well as in the sub-groups of higher performers **(B)** and lower performers **(C)** on the RBANS total scores. No negative correlations were found in the thresholded maps.

In addition to the ROI analysis based on *a priori* regions, we examined the whole-brain connectivity with regard to the PCC, as shown in Figure 2. The key regions of the DMN (PCC, mPFC, LatPar, HF and PHC) are shown with significant connectivity values, which are consistent with the above ROI analysis. A stratified analysis showed very similar patterns of DMN in the higher and lower performer groups. There are larger regions of activations in the dorsomedial prefrontal cortex (dmPFC) as well as the ventromedial prefrontal cortex (vmPFC) in the higher performers than those in the lower performers.

### 3.4 Brain-age association

**Figure 3:**
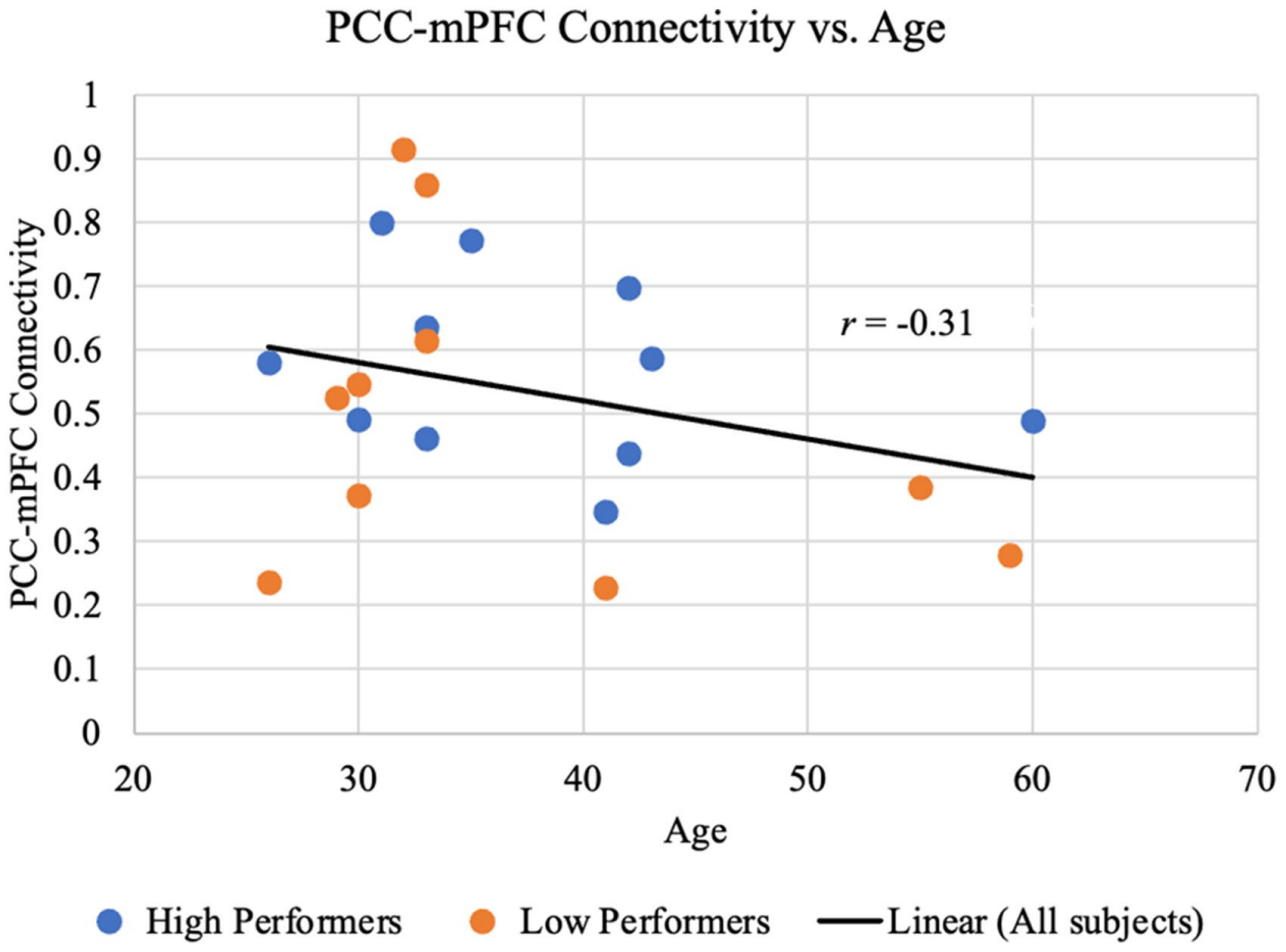
PCC-mPFC functional connectivity is not monotonically associated with age. The time course of the mPFC was correlated with the time course of the PCC for each participant. The resulting z-transformed correlation coefficient z(r) for each participant is plotted against age. Data representing lower performers on the RBANS total are colored in red, and those representing higher performers on the RBANS total are colored in blue. The black regression line, shown for illustrative purposes only, indicates an overall negative yet *not* significant trend between the PCC-mPFC functional connectivity and age (*r* = −0.31, *p* > 0.1).

Since prior studies have benchmarked the aging trajectory of the DMN connectivity, in our study we examined the relationship between age and the functional connectivity of key DMN nodes, i.e., PCC-mPFC connectivity. As shown in Figure 3, although an overall decreasing trend is observed in our study participants ranged from 26 to 60 years, the association between the PCC-mPFC connectivity and the age of years is *not* significant (*r* = −0.31, *p* > 0.1), suggesting that the age is not a strong predictor of the connectivity values. The partial correlation between PCC-mPFC connectivity and the age is not significant, either, after controlling the years of education (*r* = −0.29, *p* > 0.1). In addition, when examining the higher and lower performers separately, the age-connectivity relationship is not significant, either. Across the years, higher and lower performers spread out between the young, middle-age and older adulthood, with similar distribution of connectivity values as illustrated in Figure 3.

### 3.5 Brain-function association

**Figure 4:**
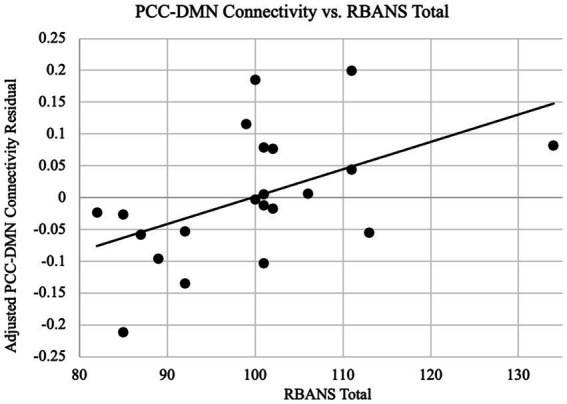
PCC-DMN connectivity are related to cognitive performance. Functional connectivity as the z-transformed correlation coefficients resulting from correlating the time courses of PCC and all other DMN nodes is plotted against the RBANS total scores, after controlling the DMN connectivity for age and gender. The regression line between the two measures is plotted on the graph. The cognitive scores are significantly associated with the PCC-DMN functional connectivity (*r* = 0.51, *p* = 0.02).

Since the connectivity-age association was not significant, we have furthermore examined the relationship between connectivity and cognitive functions as indexed by the RBANS total scores. Based on *a priori* regions of the DMN, the correlation between connectivity and the RBANS total scores for patients was found to be significant for the DMN as a whole in addition to the dmPFC specifically. As shown in Figure 4, the DMN connectivity and the RBANS total scores had a significant positive correlation coefficient (*r* = 0.51, *p* = 0.02) after controlling the DMN connectivity with age and gender, which suggests the DMN functional connectivity is a proportional indicator of individuals’ cognitive performance. The partial correlation between the DMN connectivity and the RBANS total scores remain significant, after controlling the years of education (*r* = 0.66, *p* = 0.001). The BAI and BDI were not correlated with the functional connectivity of the DMN (r_DMN_BAI = 0.18, *p* = 0.44; r_DMN_BDI = 0.04, *p* = 0.88).

**Figure 5:**
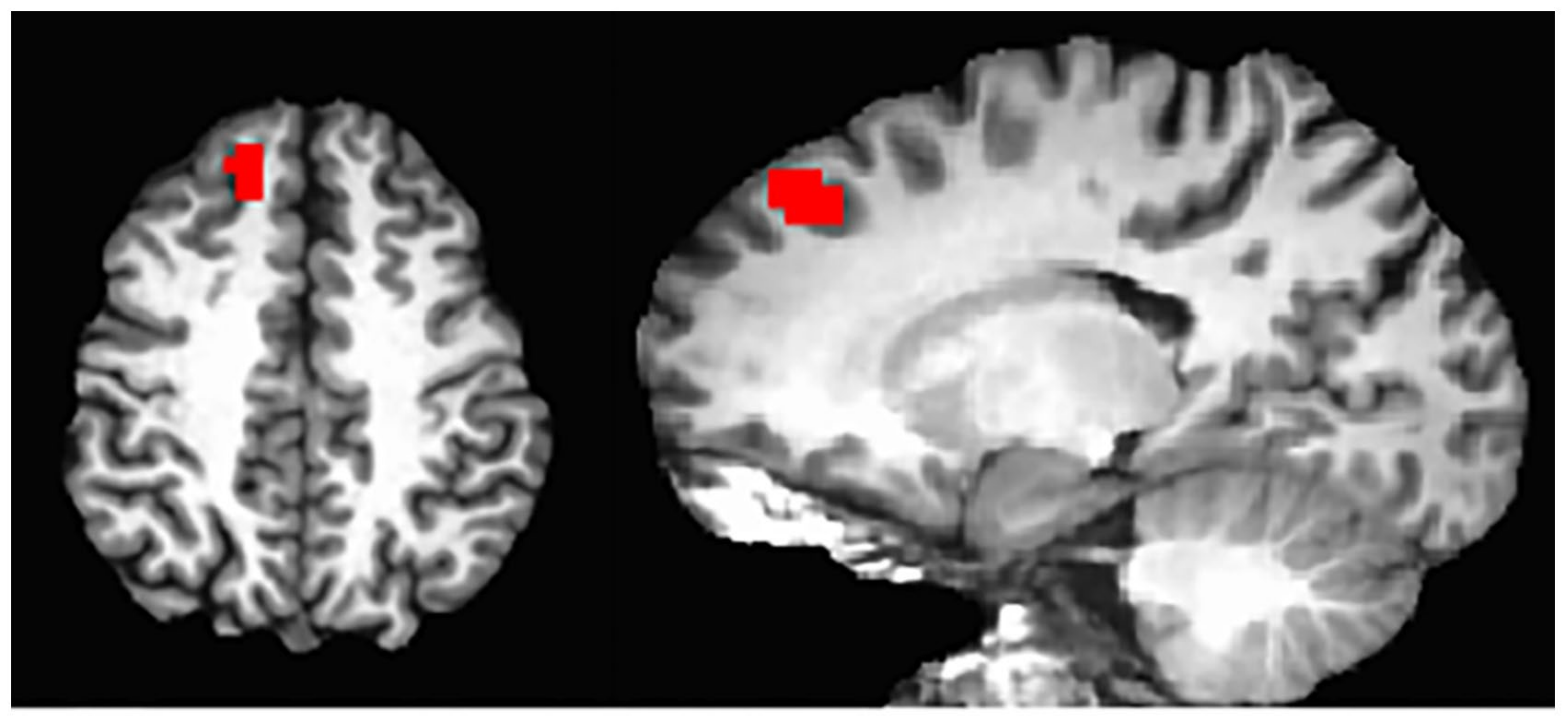
DMN connectivity covaried with RBANS total scores. Whole-brain analyses identified the regions where the resting-state functional connectivity seeded at posterior cingulate cortex is significant as well as covaried with the cognitive performance indexed by the RBANS total scores across all participants (*p*_corrected_ < 0.05), overlaid on an individual participant’s MRI. The region is a dorsal and medial portion of the PFC centered at the Talaraich coordinates of X = −16, Y = 38, Z = 41.

**Figure 6:**
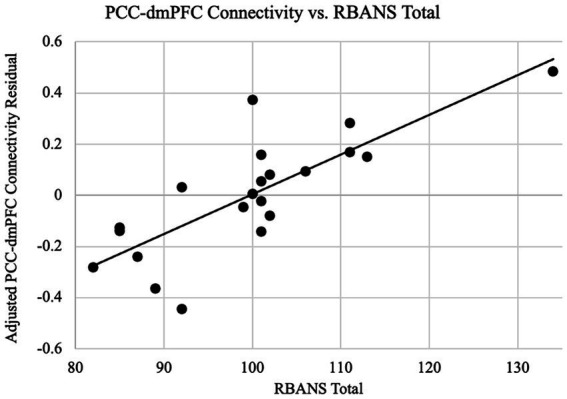
PCC-dmPFC connectivity are related to cognitive performance. Functional connectivity as the z-transformed correlation coefficients resulting from correlating the dorsal medial prefrontal cortex (dmPFC) and the PCC time courses is plotted against the RBANS total scores, after controlling the dmPFC connectivity for age and gender. The regression line between the two measures is plotted on the graph. The cognitive scores are *positively* associated with the PCC-dmPFC functional connectivity (*r* = 0.79, *p* < 0.001).

On the whole-brain level, Figure 2 shows significant connectivity in the mPFC areas, especially more extensive in the higher performers than lower performers. This data further indicated a mechanistic relationship between the mPFC and neuropsychological function in the patients. Thus, we further performed an exploratory analysis to look for areas with covariation of cognitive performance across individuals. Our results as shown in Figure 5 revealed a cluster in the frontal lobe of the brain where connectivity with PCC was significantly correlated with cognitive performance indexed by the RBANS test scores. The cluster identified a portion of the left dmPFC near Brodmann Area 9 centered at the Talaraich coordinates of X = −16, Y = 38, and Z = 41. Meanwhile, the PCC-dmPFC connectivity was correlated with the RBANS total scores with a correlation coefficient of 0.79 (*p* < 0.001) after controlling the DMN connectivity with age and gender, as shown in Figure 6. The partial correlation between the PCC-dmPFC connectivity and the RBANS total scores remain significant, after controlling the years of education (*r* = 0.82, *p* < 0.001). The PCC-dmPFC connectivity also correlated with the RBANS Index scores regarding specific cognitive domains of Immediate Memory, Delayed Memory, and Attention with correlation coefficients of 0.52 (*p* = 0.02), 0.64 (*p* = 0.002), and 0.58 (*p* = 0.008), respectively. The BAI and BDI were not correlated with the functional connectivity between PCC and dmPFC (r_dmPFC_BAI = −0.21, *p* = 0.36; r_dmPFC_BDI = 0.04, *p* = 0.86). Furthermore, the PCC-vmPFC connectivity was not revealed in the covariate analysis.

## 4 Discussion

In the current study, we have recruited a group of COVID patients who were mild, non-hospitalized cases but had subjective cognitive complaints, and we evaluated their cognitive performance and brain function using clinically validated cognitive batteries and resting state functional MRI, respectively. Although some neuroimaging studies have shown structural and functional changes in COVID, brain-network-level functional changes that underlie the memory and other cognitive domains have not yet been fully characterized in post-COVID populations, especially in a homogenous group of non-hospitalized cases. We hypothesized that patients with subjective cognitive impairment after COVID will have objective cognitive impairment on neuropsychological assessments and that their level of impairment in cognitive functions will be associated with the functional connectivity of the DMN—a functional network involved in memory and cognition. Our investigation, however, discovered that clinically significant levels of cognitive impairment did not present in the group of mild, non-hospitalized COVID patients. Nonetheless, the seed-based analysis discovered a positive correlation between the DMN connectivity and cognitive performance. Additionally, the whole-brain analysis discovered a specific connection between PCC and dmPFC that was positively correlated with the RBANS scores as well.

### 4.1 Neurocognitive findings

Surprisingly, objective cognitive impairment was *not* present within this study group as a whole, despite subjects’ complaints. According to the clinical evaluations, both the RBANS and MoCA scores of the study group have not yielded significant levels of cognitive impairment as compared to a population-based normative scores, indicating a lack of apparent general objective cognitive decline from mild COVID. Still, the indication between severe COVID and related neurocognitive impairment has been discussed and reviewed in multiple studies (Almeria et al., 2020; Fisicaro et al., 2021; Iadecola et al., 2020; Jha et al., 2021; Lu et al., 2020; Mao et al., 2020; Miners et al., 2020; Najjar et al., 2020; Rogers et al., 2020; Varatharaj et al., 2020). Notably, none of our subjects had any hospitalization or admission to ICU, and many reports of long-COVID admittedly result from more severe COVID cases (Del Brutto et al., 2021). The fact that our study does not find cognitive impairment detectable by general neuropsychological examination could be due to the mild severity of COVID experienced by the subjects, or even the generally long duration of 9.45 months on average since COVID contraction within the cohort. However, a phenomenon supported by previous studies would point at the ‘diagnostic threat’ or psychological distress resulting from COVID as the likely cause (Gouraud et al., 2021; Winter and Braw, 2023). Individuals who are diagnosed with the virus and recognize the resulting risk of impairment can experience distress that detracts from day-to-day functioning. Though this detraction can be perceived as cognitive impairment, it differs from the objective impairment presented in previous studies. While our study protocol has excluded subjects with moderate anxiety or depression, again, clinically significant cognitive impairment was not present within this study group as a whole. A substantial variability is observed, such that the lower performers as a subgroup significantly differed in the cognitive assessment than those upper performers, as summarized in Table 1. The occurrence of three out of 21 participants scoring below 85 is consistent with what would be expected in the normal population in this age range (Randolph et al., 1998). Nonetheless, an individual’s score within the range of normative scores is not entirely contradictive to meaning a significant decline of cognitive performance since these normative scores are generated on a population basis (Randolph et al., 1998). Since we only evaluated the subjects once, the subjective complaint of memory decline may still be valid, but investigation would require data to compare the cognitive performance before and after COVID.

### 4.2 Default mode network

Our results of the post-COVID subjects with cognitive complaints have replicated the organization of the DMN reported in healthy populations in the adulthood. As shown in Figure 1, the key nodes of the DMN have been preserved, presenting significant group-level connectivity. Since the age range of our recruited subjects is from 26 to 60 years, such finding is consistent with previous reports of the DMN in the normal healthy population in adulthood (Andrews-Hanna et al., 2007; Ball et al., 2017; Chiesa et al., 2019; Staffaroni et al., 2018; Zonneveld et al., 2019). Prior studies on the DMN have reported disconnection of the DMN or significant loss of connectivity between PCC and mPFC only in the stage of advanced aging (e.g., in elderly older than 60 years; Andrews-Hanna et al., 2007; Damoiseaux et al., 2008), or in the Alzheimer’s Disease Related Dementia (Greicius et al., 2004). As our participants have performed near the expected level on general neuropsychological examinations, it is expected that the DMN still demonstrates strong connectivity.

Noteworthy, the measured connectivity values within this study were actually higher than healthy controls of a similar age presented in the study by Andrews-Hanna et al. (2007). This difference of values could primarily be attributed to differences in the magnetic field strength of the MRI scanners used. Within our study group, data was obtained using a 3 T GE Scanner while the data of comparable healthy controls was obtained using a 1.5 T Siemens Scanner (Andrews-Hanna et al., 2007). Previous studies indicate both greater signal output and contrast to noise ratios from higher magnetic field strength scanners (Okada et al., 2005). Such increases in signal and relative decreases in noise would greatly benefit the correlation between fMRI time series used to calculate the functional connectivity. The similarities in the DMN connectivity between this group and normative controls point to the preservation of the DMN in non-hospitalized post-COVID individuals with subjective cognitive complaints.

### 4.3 Brain-age association

Despite that our data have identified the DMN in non-hospitalized post-COVID individuals, our study did *not* find any direct age association in the functional connectivity of the DMN, as summarized in Figure 3. Previous studies have indicated decreases in the DMN connectivity coinciding with increased age, even in a linear-approaching manner in the elderly range of 60 years old and above (Andrews-Hanna et al., 2007). This decreasing trend presented in Figure 3, but the Pearson correlation coefficient relating age to the PCC-mPFC connectivity was *not* significant (*r* = −0.31, *p* > 0.1). This is because our study sample includes participants in their adulthood from 26 to 60 years old, while the studies that reported a linear aging-related deduction of the DMN connectivity had much older subjects (60 years old above). Also, our sample size has limited power to find an association with age. Nonetheless, cognitive function is known to decrease alongside age and the DMN connectivity (Buckner et al., 2008; Mevel et al., 2011). Thus, our investigation was shifted to the brain-functional association.

### 4.4 Brain-function association

Importantly, a remarkable new finding in our study is a direct brain-function association, especially a significant positive correlation between the DMN connectivity and the level of cognitive performance. So far there is limited data surrounding neurocognitive outcomes in COVID patients. Although some studies have shown structural and functional changes in patients with COVID (Fischer et al., 2020; Esposito et al., 2022; Zhang et al., 2022; Díez-Cirarda et al., 2023; Muccioli et al., 2023), our neuroimaging findings in a homogenous group of mild, non-hospitalized COVID patients with cognitive self-complaints are an important addition to the existing literature. Concerning the DMN, a well-established network that underlies the normal memory function (Andrews-Hanna et al., 2007) and a signature in the AD related impairment (Sorg et al., 2007; Petrella et al., 2011), we found that the connectivity within the key nodes of the DMN was strongly correlated with the RBANS performance with a *positive* correlation of 0.52 (*p* = 0.02), shown in Figure 4. Moreover, through an exploratory, whole-brain analysis, we also found that the dmPFC, another key region for the advanced human cognition (Eickhoff et al., 2016), presents connectivity as an even stronger indicator of cognitive performance with a correlation coefficient of r = 0.79 (*p* < 0.001), shown in Figures 5, 6. The trend that higher performance is associated with higher connectivity is consistent with other studies reported in COVID subjects (Díez-Cirarda et al., 2023; Fineschi et al., 2024). While the study by Díez-Cirarda et al., 2023 included both hospitalized and non-hospitalized COVID cases, our sample only pertains to the mild, non-hospitalized individuals, emphasizing DMN’s role in their cognitive performance. Meanwhile, a recent study by Fineschi et al. (2024) also studied non-hospitalized post-COVID with cognitive complaints and reported positive correlation between post-COVID symptoms and increased connectivity in the right temporoparietal junction, which is part of the DMN as observed in our study. However, the Fineschi et al. (2024) study did *not* find any significant correlation between the cognitive functions and the network connectivity as our investigation did. Importantly, our analysis utilized a prior seed defined DMN, which differed from the completely data-driven approach in the Fineschi et al. study. Our findings are overall consistent with previous studies on cognitive function in healthy populations as well as the cognitive decline in normal aging, where higher performance is associated with higher connectivity in the DMN and decreases of its connectivity understandably correspond to decreases in the related cognitive functions (Andrews-Hanna et al., 2007; Damoiseaux et al., 2008). Considering the DMN as a meaningful neuroimaging-based indictor of the normal cognitive status, our finding of the positive association suggests that the mild COVID has not changed the status of the DMN, although there are complaints or even possibly individual-level impairment.

The positive association of brain connectivity as found in our results deviates from a reversed, negative association such that lower performance is related to elevated connectivity—a phenomena that has been reported in mild cognitive impairment related to prodromal stage of Alzheimer’s Disease (Sorg et al., 2007; Damoiseaux et al., 2012; De Vogelaere et al., 2012; Gardini et al., 2015; Chiesa et al., 2019). Studies on neurodegenerative diseases have presented a type of compensatory mechanism, where cognitive decline is associated with increasing levels of connectivity (Amieva et al., 2014; Kanishka Jha, 2023). As brain functions decrease, altered recruitment and networking can result in increasing levels of connectivity and activation despite decreased performance. For example, the PCC and other key nodes of the DMN have been shown to preserve higher resting state functional connectivity in individuals with mild cognitive impairment that did not convert to dementia rather than those whose impairment did convert to dementia, suggesting the compensatory role of PCC against deterioration (Petrella et al., 2011). Another example is the hippocampus and related structures within the medial temporal lobe and prefrontal cortices, where low-performing older adults (~60 years or older) demonstrated greater activation to achieve successful encoding trials (Miller et al., 2008), possibly as a compensatory response. Although this phenomenon has been discovered in AD-related cognitive impairment, our study of post-COVID individuals indicate that such a compensatory mechanism is *not* present in the DMN. However, our study sample did present a higher connectivity involving the hippocampal formation and the parahippocampal cortex in those lower performers, which is consistent with recent report in post-COVID individuals presenting cognitive impairment (Dacosta-Aguayo et al., 2024), as well as consistently observed in elderly normal (Miller et al., 2008) and mild cognitive impairment (Gardini et al., 2015). Interestingly, the hippocampal compensatory response is observed in our sample of adult participants with average age of 37 years, which is 20 years or more younger than other studies (Gardini et al., 2015; Miller et al., 2008). The implication of such negative association, however, warrants further studies.

Additionally, our study has identified the dmPFC exhibiting strong functional connectivity with the PCC and a significant positive correlation with the RBANS total scores. Furthermore, this connectivity was correlated with subtest performance on the RBANS Index scores regarding Immediate Memory and Delayed Memory, indicating better memory performance among those with higher PCC-dmPFC connectivity. Memory loss was a commonly reported symptom from those included in the study and vital to examine post COVID recovery. The correlation exhibited between the dmPFC and the RBANS performance aligns with previous findings in normal aging individuals (Cajanus et al., 2019; Friedman and Robbins, 2022), indicating the possibility of a similar phenomenon in individuals complaining of neurologic symptoms. In addition, strong correlation was also demonstrated between the PCC-dmPFC connection and the RBANS Index score regarding attention. As a significant symptom of neuropsychological decay, attention is associated with the Brodmann Area 9 and PFC (Szczepanski and Knight, 2014). These associations notably draw parallels to the phenomena observed again in normal aging-related declines, despite a lack of clinical impairment through the neuropsychological testing.

Although the trends in the DMN exhibited in this group of post-COVID participants are similar to the correlations found with age-related decline (Andrews-Hanna et al., 2007; Damoiseaux et al., 2008), there is a remarkable age difference (adult participants in our study vs. elderly participants in others). Inflammatory changes and hypoxic brain injury have been proposed as potential mechanisms of neurologic dysfunction in COVID patients, both of which have been described in the pathophysiology of AD (Iadecola et al., 2020; Miners et al., 2020). Additionally, structural findings have pointed to CSF immune dysregulation that results in the proposed inflammation and ischemia (Fernández-Castañeda et al., 2022; Mina et al., 2023). Prior studies of rs-fMRI in AD and mild cognitive impairment have also demonstrated correlations between mini-mental state exam (MMSE) scores and network connectivity (Li et al., 2020). Olfactory nerve invasion by SARS COV-2 has also been hypothesized as a potential mechanism of anosmia and has been frequently implicated in COVID patients with neurologic impairment (Jha et al., 2021). While the findings herein do not directly validate such mechanisms, the positive correlation between the DMN connectivity and cognitive performance does point toward a neural decay possibly through inflammation while pointing away from the possibility of a compensatory mechanism outside of the HF-PHC connection. Preliminary findings have discovered irregularities in CSF immune function corresponding with neurocirculatory inflammation and ischemia that were proposed by many as potential mechanisms for reported cognitive decay resulting from COVID (Fernández-Castañeda et al., 2022; Mina et al., 2023). Importantly, however, it is *unclear* whether COVID may pose an increased risk for early-onset dementia or cognitive impairment in patients with additional risk factors (Manzo et al., 2021). Thus, though these mechanisms may likely present during and beyond COVID, further examinations are necessary in more severe cases. Additional measures such as biomarker of inflammatory changes and hypoxic brain injury would be needed to provide insights to the underlying mechanism.

The symptoms of depression and anxiety have been shown to impact the brain networks in patients with COVID (Benedetti et al., 2021), as anxiety and depression have also been shown to affect the DMN connectivity in patients with affective disorders (Drevets et al., 2008; Greicius et al., 2007; Sheline et al., 2010). In our study design, we used BAI and BDI to manage anxiety and depression scales as confounding factors for our investigation of the DMN connectivity, respectively, by excluding patients with anxiety/depression conditions. However, meta-analysis has revealed that anxiety is a common symptom (7–46%) reported in non-hospitalized patients in the postacute (>4 weeks, >3–6 months) period after COVID (Frontera and Simon, 2022). Therefore, our sample may be skewed toward a population of non-hospitalized COVID that do not have comorbid anxiety or depression, while future comprehensive studies of COVID that include those patients with anxiety or depression are warranted to have a representation of a general population.

### 4.5 Limitations

Though this study has made novel findings regarding the mild, non-hospitalized COVID participants, it is limited in several ways. Primarily, its small sample size limits the relation between this group and the population as a whole. Also, the small sample size limited our power to detect the association between brain connectivity and age. We did not include healthy control participants to be studied using the identical methodology of clinical batteries and neuroimaging, which could attest to whether the association that our study has discovered in the mild COVID patients would be any different from that in a healthy control group, attributed to other factors such as aging or as a sequela of any viral or bacterial infection. Additionally, although the RBANS battery is especially suited for repeated test, our study has only acquired one-time only assessment. Future efforts are warranted to compare with the baseline prior to COVID (Douaud et al., 2022), as well as to study the long-term deterioration of cognitive decline in post-COVID subjects (Invernizzi et al., 2024), and even through the comparison with the aging trajectory of healthy controls. Longitudinal information for comparison would be greatly beneficial in revealing the specific alterations in the connectivity of specific brain regions after COVID.

## 5 Conclusion

This study examined functional connectivity via resting state fMRI in a group of mild, non-hospitalized COVID participants suffering from cognitive complaints to provide insight into the phenomena and mechanism by which this apparent brain fog occurs. Our investigation discovered a positive correlation between the DMN connectivity and cognitive performance, but clinically significant levels of cognitive impairment did not present through neuropsychological testing. Additionally, the findings also discovered a specific connection between PCC and dmPFC that was greatly correlated with the RBANS scores, and this connection between DMN or dmPFC has often been found to be altered in forms of age-related cognitive decline. Our results again stressed the important role of DMN and dmPFC underlying cognitive performance, especially here in our study of non-hospitalized COVID participants. Importantly, the positive correlation in DMN and dmPFC is possibly indicative of a neural decay mechanism, as opposed to a compensatory mechanism otherwise. This study has provided a novel insight into the functional effects among a group of individuals reporting cognitive complaints after non-hospitalized COVID, but further functional studies in more severe cases of COVID and longitudinal studies with repeated cognitive and neuroimaging evaluations may be warranted.

## Data Availability

The raw data supporting the conclusions of this article will be made available by the authors, without undue reservation.

## References

[ref1] Almeria M. Cejudo J. C. Sotoca J. Deus J. Krupinski J. (2020). Cognitive profile following COVID-19 infection: clinical predictors leading to neuropsychological impairment. Brain Behav Immun Health 9:100163. doi: 10.1016/j.bbih.2020.100163, PMID: 33111132 PMC7581383

[ref2] Amieva H. Mokri H. Le Goff M. Meillon C. Jacqmin-Gadda H. Foubert-Samier A.. (2014). Compensatory mechanisms in higher-educated subjects with Alzheimer’s disease: a study of 20 years of cognitive decline. Brain 137, 1167–1175. doi: 10.1093/brain/awu035, PMID: 24578544

[ref3] Amodio D. M. Frith C. D. (2006). Meeting of minds: the medial frontal cortex and social cognition. Nat. Rev. Neurosci. 7, 268–277. doi: 10.1038/nrn1884, PMID: 16552413

[ref4] Andrews-Hanna J. R. Snyder A. Z. Vincent J. L. Lustig C. Head D. Raichle M. E.. (2007). Disruption of large-scale brain systems in advanced aging. Neuron 56, 924–935. doi: 10.1016/j.neuron.2007.10.038 18054866 PMC2709284

[ref5] Ball G. Beare R. Seal M. L. (2017). Network component analysis reveals developmental trajectories of structural connectivity and specific alterations in autism spectrum disorder. Hum. Brain Mapp. 38, 4169–4184. doi: 10.1002/hbm.23656, PMID: 28560746 PMC6866922

[ref6] Behrmann M. Geng J. J. Shomstein S. (2004). Parietal cortex and attention. Curr. Opin. Neurobiol. 14, 212–217. doi: 10.1016/j.conb.2004.03.012, PMID: 15082327

[ref7] Benedetti F. Palladini M. Paolini M. Melloni E. Vai B. De Lorenzo R.. (2021). Brain correlates of depression, post-traumatic distress, and inflammatory biomarkers in COVID-19 survivors: a multimodal magnetic resonance imaging study. Brain Behav Immun Health 18:100387. doi: 10.1016/j.bbih.2021.100387, PMID: 34746876 PMC8562046

[ref8] Benge J. F. Kiselica A. M. (2021). Rapid communication: preliminary validation of a telephone adapted Montreal cognitive assessment for the identification of mild cognitive impairment in Parkinson's disease. Clin. Neuropsychol. 35, 133–147. doi: 10.1080/13854046.2020.1801848, PMID: 32779959

[ref9] Biswal B. Zerrin Yetkin F. Haughton V. M. Hyde J. S. (1995). Functional connectivity in the motor cortex of resting human brain using echo-planar mri. Magn. Reson. Med. 34, 537–541. doi: 10.1002/mrm.1910340409, PMID: 8524021

[ref10] Buckner R. L. Andrews-Hanna J. R. Schacter D. L. (2008). The brain's default network: anatomy, function, and relevance to disease. Ann. N. Y. Acad. Sci. 1124, 1–38. doi: 10.1196/annals.1440.011, PMID: 18400922

[ref11] Buckner R. L. Snyder A. Z. Shannon B. J. LaRossa G. Sachs R. Fotenos A. F.. (2005). Molecular, structural, and functional characterization of Alzheimer's disease: evidence for a relationship between default activity, amyloid, and memory. J. Neurosci. 25, 7709–7717. doi: 10.1523/JNEUROSCI.2177-05.2005, PMID: 16120771 PMC6725245

[ref12] Cajanus A. Solje E. Koikkalainen J. Lötjönen J. Suhonen N.-M. Hallikainen I.. (2019). The association between distinct frontal brain volumes and behavioral symptoms in mild cognitive impairment, Alzheimer's disease, and frontotemporal dementia. Front. Neurol. 10. doi: 10.3389/fneur.2019.01059 PMC6786130 31632342

[ref13] Chen Y. Tang J. H. De Stefano L. A. Wenger M. J. Ding L. Craft M. A.. (2022). Electrophysiological resting state brain network and episodic memory in healthy aging adults. NeuroImage 253:118926. doi: 10.1016/j.neuroimage.2022.118926 35066158

[ref14] Chiesa P. A. Cavedo E. Vergallo A. Lista S. Potier M. C. Habert M. O.. (2019). Differential default mode network trajectories in asymptomatic individuals at risk for Alzheimer's disease. Alzheimers Dement. 15, 940–950. doi: 10.1016/j.jalz.2019.03.006, PMID: 31113760

[ref15] Collinson S. L. Fang S. H. Lim M. L. Feng L. Ng T. P. (2014). Normative data for the repeatable battery for the assessment of neuropsychological status in elderly Chinese. Arch. Clin. Neuropsychol. 29, 442–455. doi: 10.1093/arclin/acu023, PMID: 24903208

[ref16] Coronavirus and the Nervous System (2023). National Institute of Neurological Disorders and Stroke. U.S: Department of Health and Services.

[ref17] Dacosta-Aguayo R. Torán-Monserrat P. Carmona-Cervelló M. León-Gómez B. B. Mataró M. Puig J.. (2024). Multimodal neuroimaging in long-COVID and its correlates with cognition 1.8 years after SARS-CoV-2 infection: a cross-sectional study of the Aliança ProHEpiC-19 Cognitiu. Front. Neurol. 15:1426881. doi: 10.3389/fneur.2024.1426881 39346769 PMC11428557

[ref18] Damoiseaux J. S. Beckmann C. F. Arigita E. J. Barkhof F. Scheltens P. Stam C. J.. (2008). Reduced resting-state brain activity in the "default network" in normal aging. Cereb. Cortex 18, 1856–1864. doi: 10.1093/cercor/bhm207, PMID: 18063564

[ref19] Damoiseaux J. S. Prater K. E. Miller B. L. Greicius M. D. (2012). Functional connectivity tracks clinical deterioration in Alzheimer's disease. Neurobiol. Aging 33, 828.e19–828.e30. doi: 10.1016/j.neurobiolaging.2011.06.024, PMID: 21840627 PMC3218226

[ref20] De Vogelaere F. Santens P. Achten E. Boon P. Vingerhoets G. (2012). Altered default-mode network activation in mild cognitive impairment compared with healthy aging. Neuroradiology 54, 1195–1206. doi: 10.1007/s00234-012-1036-6, PMID: 22527687

[ref21] Del Brutto O. H. Wu S. Mera R. M. Costa A. F. Recalde B. Y. Issa N. P. (2021). Cognitive decline among individuals with history of mild symptomatic SARS-CoV-2 infection. A longitudinal prospective study nested to a population cohort. Eur. J. Neurol. 28, 3245–3253. doi: 10.1111/ene.14775 33576150 PMC8014083

[ref22] Díez-Cirarda M. Yus M. Gómez-Ruiz N. Polidura C. Gil-Martínez L. Delgado-Alonso C.. (2023). Multimodal neuroimaging in post-COVID syndrome and correlation with cognition. Brain 146, 2142–2152. doi: 10.1093/brain/awac384, PMID: 36288544 PMC9620345

[ref23] Douaud G. Lee S. Alfaro-Almagro F. Arthofer C. Wang C. McCarthy P.. (2022). SARS-CoV-2 is associated with changes in brain structure in UK biobank. Nature 604, 697–707. doi: 10.1038/s41586-022-04569-5, PMID: 35255491 PMC9046077

[ref24] Drevets W. C. Price J. L. Furey M. L. (2008). Brain structural and functional abnormalities in mood disorders: implications for neurocircuitry models of depression. Brain Struct. Funct. 213, 93–118. doi: 10.1007/s00429-008-0189-x, PMID: 18704495 PMC2522333

[ref25] Egbert A. R. Cankurtaran S. Karpiak S. (2020). Brain abnormalities in COVID-19 acute/subacute phase: a rapid systematic review. Brain Behav. Immun. 89, 543–554. doi: 10.1016/j.bbi.2020.07.014, PMID: 32682993 PMC7366124

[ref26] Eickhoff S. B. Laird A. R. Fox P. T. Bzdok D. Hensel L. (2016). Functional segregation of the human dorsomedial prefrontal cortex. Cereb. Cortex 26, 304–321. doi: 10.1093/cercor/bhu250, PMID: 25331597 PMC4677979

[ref27] Esposito F. Cirillo M. De Micco R. Caiazzo G. Siciliano M. Russo A. G.. (2022). Olfactory loss and brain connectivity after COVID-19. Hum. Brain Mapp. 43, 1548–1560. doi: 10.1002/hbm.25741, PMID: 35083823 PMC8886650

[ref28] Fernández-Castañeda A. Lu P. Geraghty A. C. Song E. Lee M. H. Wood J.. (2022). Mild respiratory COVID can cause multi-lineage neural cell and myelin dysregulation. Cell 185, 2452–2468.e16. doi: 10.1016/j.cell.2022.06.008, PMID: 35768006 PMC9189143

[ref29] Fineschi S. Fahlström M. Fällmar D. Haller S. Wikström J. (2024). Comprehensive MRI assessment reveals subtle brain findings in non-hospitalized post-COVID patients with cognitive impairment. Front. Neurosci. 18:1435218. doi: 10.3389/fnins.2024.1435218, PMID: 39319311 PMC11420131

[ref30] Fischer D. Threlkeld Z. D. Bodien Y. G. Kirsch J. E. Huang S. Y. Schaefer P. W.. (2020). Intact brain network function in an unresponsive patient with COVID-19. Ann. Neurol. 88, 851–854. doi: 10.1002/ana.25838, PMID: 32613682 PMC7361474

[ref31] Fisicaro F. Di Napoli M. Liberto A. Fanella M. Di Stasio F. Pennisi M.. (2021). Neurological sequelae in patients with COVID-19: a histopathological perspective. Int. J. Environ. Res. Public Health 18:1415. doi: 10.3390/ijerph18041415, PMID: 33546463 PMC7913756

[ref32] Fox M. D. Snyder A. Z. Vincent J. L. Corbetta M. Van Essen D. C. Raichle M. E. (2005). The human brain is intrinsically organized into dynamic, anticorrelated functional networks. Proc. Natl. Acad. Sci. USA 102, 9673–9678. doi: 10.1073/pnas.0504136102, PMID: 15976020 PMC1157105

[ref33] Fox M. D. Zhang D. Snyder A. Z. Raichle M. E. (2009). The global signal and observed anticorrelated resting state brain networks. J. Neurophysiol. 101, 3270–3283. doi: 10.1152/jn.90777.2008, PMID: 19339462 PMC2694109

[ref34] Friedman N. P. Robbins T. W. (2022). The role of prefrontal cortex in cognitive control and executive function. Neuropsychopharmacology 47, 72–89. doi: 10.1038/s41386-021-01132-0, PMID: 34408280 PMC8617292

[ref35] Frontera J. A. Simon N. M. (2022). Bridging knowledge gaps in the diagnosis and Management of Neuropsychiatric Sequelae of COVID-19. JAMA Psychiatry 79, 811–817. doi: 10.1001/jamapsychiatry.2022.1616, PMID: 35767287

[ref36] Gardini S. Venneri A. Sambataro F. Cuetos F. Fasano F. Marchi M.. (2015). Increased functional connectivity in the default mode network in mild cognitive impairment: a maladaptive compensatory mechanism associated with poor semantic memory performance. J. Alzheimers Dis. 45, 457–470. doi: 10.3233/JAD-142547, PMID: 25547636

[ref37] Gouraud C. Bottemanne H. Lahlou-Laforêt K. Blanchard A. Günther S. Batti S. E.. (2021). Association between psychological distress, cognitive complaints, and neuropsychological status after a severe COVID-19 episode: a cross-sectional study. Front. Psychol. 12:725861. doi: 10.3389/fpsyt.2021.725861 PMC8446522 34539470

[ref38] Greicius M. D. Flores B. H. Menon V. Glover G. H. Solvason H. B. Kenna H.. (2007). Resting-state functional connectivity in major depression: abnormally increased contributions from subgenual cingulate cortex and thalamus. Biol. Psychiatry 62, 429–437. doi: 10.1016/j.biopsych.2006.09.020, PMID: 17210143 PMC2001244

[ref39] Greicius M. D. Srivastava G. Reiss A. L. Menon V. (2004). Default-mode network activity distinguishes Alzheimer's disease from healthy aging: evidence from functional MRI. Proc. Natl. Acad. Sci. USA 101, 4637–4642. doi: 10.1073/pnas.0308627101, PMID: 15070770 PMC384799

[ref40] Hampshire A. Trender W. Chamberlain S. R. Jolly A. E. Grant J. E. Patrick F.. (2021). Cognitive deficits in people who have recovered from COVID-19. EClinicalMedicine 39:101044. doi: 10.1016/j.eclinm.2021.101044, PMID: 34316551 PMC8298139

[ref42] Iadecola C. Anrather J. Kamel H. (2020). Effects of COVID-19 on the nervous system. Cell 183:e11, 16–27.e1. doi: 10.1016/j.cell.2020.08.028, PMID: 32882182 PMC7437501

[ref43] Invernizzi A. Renzetti S. van Thriel C. Rechtman E. Patrono A. Ambrosi C.. (2024). COVID-19 related cognitive, structural and functional brain changes among Italian adolescents and young adults: a multimodal longitudinal case-control study. Transl. Psychiatry 14:402. doi: 10.1038/s41398-024-03108-2, PMID: 39358346 PMC11447249

[ref44] Jha N. K. Ojha S. Jha S. K. Dureja H. Singh S. K. Shukla S. D.. (2021). Evidence of coronavirus (CoV) pathogenesis and emerging pathogen SARS-CoV-2 in the nervous system: a review on neurological impairments and manifestations. J. Mol. Neurosci. 71, 2192–2209. doi: 10.1007/s12031-020-01767-6, PMID: 33464535 PMC7814864

[ref45] Kanishka Jha S. K. (2023). Compensatory cognition in neurological diseases and aging: a review of animal and human studies. Aging Brain 3:100061. doi: 10.1016/j.nbas.2022.100061, PMID: 36911258 PMC9997140

[ref47] Li X. Wang F. Liu X. Cao D. Cai L. Jiang X.. (2020). Changes in brain function networks in patients with amnestic mild cognitive impairment: a resting-state fMRI study. Front. Neurol. 11:554032. doi: 10.3389/fneur.2020.554032 33101173 PMC7554345

[ref48] Lilliefors H. W. (1967). On the Kolmogorov-Smirnov test for normality with mean and variance unknown. J. Am. Stat. Assoc. 62, 399–402. doi: 10.1080/01621459.1967.10482916

[ref49] Lu Y. Li X. Geng D. Mei N. Wu P. Y. Huang C. C.. (2020). Cerebral Micro-structural changes in COVID-19 patients - an MRI-based 3-month follow-up study. EClinicalMedicine 25:100484. doi: 10.1016/j.eclinm.2020.100484, PMID: 32838240 PMC7396952

[ref50] Manzo C. Serra-Mestres J. Isetta M. Castagna A. (2021). Could COVID-19 anosmia and olfactory dysfunction trigger an increased risk of future dementia in patients with ApoE4? Med. Hypotheses 147:110479. doi: 10.1016/j.mehy.2020.110479, PMID: 33422806 PMC7785277

[ref51] Mao L. Jin H. Wang M. Hu Y. Chen S. He Q.. (2020). Neurologic manifestations of hospitalized patients with coronavirus disease 2019 in Wuhan, China. JAMA Neurol. 77, 683–690. doi: 10.1001/jamaneurol.2020.1127, PMID: 32275288 PMC7149362

[ref52] Mevel K. Chételat G. Eustache F. Desgranges B. (2011). The default mode network in healthy aging and Alzheimer's disease. Int. J. Alzheimers Dis. 2011:535816. doi: 10.4061/2011/535816, PMID: 21760988 PMC3132539

[ref53] Miller S. L. Celone K. DePeau K. Diamond E. Dickerson B. C. Rentz D.. (2008). Age-related memory impairment associated with loss of parietal deactivation but preserved hippocampal activation. Proc. Natl. Acad. Sci. 105, 2181–2186. doi: 10.1073/pnas.0706818105, PMID: 18238903 PMC2538895

[ref54] Mina Y. Enose-Akahata Y. Hammoud D. A. Videckis A. J. Narpala S. R. O'Connell S. E.. (2023). Deep phenotyping of neurologic Postacute sequelae of SARS-CoV-2 infection. Neurol Neuroimmunol Neuroinflamm 10:e200097. doi: 10.1212/NXI.0000000000200097, PMID: 37147136 PMC10162706

[ref55] Miners S. Kehoe P. G. Love S. (2020). Cognitive impact of COVID-19: looking beyond the short term. Alzheimers Res. Ther. 12:170. doi: 10.1186/s13195-020-00744-w, PMID: 33380345 PMC7772800

[ref56] Muccioli L. Sighinolfi G. Mitolo M. Ferri L. Jane Rochat M. Pensato U.. (2023). Cognitive and functional connectivity impairment in post-COVID-19 olfactory dysfunction. Neuroimage Clin 38:103410. doi: 10.1016/j.nicl.2023.103410, PMID: 37104928 PMC10165139

[ref57] Murphy K. Birn R. M. Handwerker D. A. Jones T. B. Bandettini P. A. (2009). The impact of global signal regression on resting state correlations: are anti-correlated networks introduced? NeuroImage 44, 893–905. doi: 10.1016/j.neuroimage.2008.09.036, PMID: 18976716 PMC2750906

[ref58] Najjar S. Najjar A. Chong D. J. Pramanik B. K. Kirsch C. Kuzniecky R. I.. (2020). Central nervous system complications associated with SARS-CoV-2 infection: integrative concepts of pathophysiology and case reports. J. Neuroinflammation 17:231. doi: 10.1186/s12974-020-01896-0, PMID: 32758257 PMC7406702

[ref59] Nasreddine Z. S. Phillips N. A. Bédirian V. Charbonneau S. Whitehead V. Collin I.. (2005). The Montreal cognitive assessment, MoCA: a brief screening tool for mild cognitive impairment. J. Am. Geriatr. Soc. 53, 695–699. doi: 10.1111/j.1532-5415.2005.53221.x, PMID: 15817019

[ref60] Natu V. S. Lin J.-J. Burks A. Arora A. Rugg M. D. Lega B. (2019). Stimulation of the posterior cingulate cortex impairs episodic memory encoding. J. Neurosci. 39, 7173–7182. doi: 10.1523/JNEUROSCI.0698-19.2019, PMID: 31358651 PMC6733540

[ref61] Okada T. Yamada H. Ito H. Yonekura Y. Sadato N. (2005). Magnetic field strength increase yields significantly greater contrast-to-noise ratio increase: measured using BOLD contrast in the primary visual area. Acad. Radiol. 12, 142–147. doi: 10.1016/j.acra.2004.11.012 15721590

[ref62] Öner M. Deveci Kocakoç İ. (2017). JMASM 49: a compilation of some popular goodness of fit tests for normal distribution: their algorithms and MATLAB codes (MATLAB). J. Mod. Appl. Stat. Methods 16, 547–575. doi: 10.22237/jmasm/1509496200, PMID: 30393468

[ref63] Petrella J. R. Sheldon F. C. Prince S. E. Calhoun V. D. Doraiswamy P. M. (2011). Default mode network connectivity in stable vs progressive mild cognitive impairment. Neurology 76, 511–517. doi: 10.1212/WNL.0b013e31820af94e, PMID: 21228297 PMC3053179

[ref64] Race E. Keane M. M. Verfaellie M. (2011). Medial temporal lobe damage causes deficits in episodic memory and episodic future thinking not attributable to deficits in narrative construction. J. Neurosci. 31, 10262–10269. doi: 10.1523/JNEUROSCI.1145-11.2011, PMID: 21753003 PMC4539132

[ref65] Raichle M. E. MacLeod A. M. Snyder A. Z. Powers W. J. Gusnard D. A. Shulman G. L. (2001). A default mode of brain function. Proc. Natl. Acad. Sci. USA 98, 676–682. doi: 10.1073/pnas.98.2.676, PMID: 11209064 PMC14647

[ref66] Randolph C. Tierney M. C. Mohr E. Chase T. N. (1998). The repeatable battery for the assessment of neuropsychological status (RBANS): preliminary clinical validity. J. Clin. Exp. Neuropsychol. 20, 310–319. doi: 10.1076/jcen.20.3.310.823, PMID: 9845158

[ref67] Rivera-Izquierdo M. Láinez-Ramos-Bossini A. J. de Alba I. G. Ortiz-González-Serna R. Serrano-Ortiz Á. Fernández-Martínez N. F.. (2022). Long COVID 12 months after discharge: persistent symptoms in patients hospitalised due to COVID-19 and patients hospitalised due to other causes-a multicentre cohort study. BMC Med. 20:92. doi: 10.1186/s12916-022-02292-6, PMID: 35193574 PMC8863509

[ref68] Rogers J. P. Chesney E. Oliver D. Pollak T. A. McGuire P. Fusar-Poli P.. (2020). Psychiatric and neuropsychiatric presentations associated with severe coronavirus infections: a systematic review and meta-analysis with comparison to the COVID-19 pandemic. Lancet Psychiatry 7, 611–627. doi: 10.1016/S2215-0366(20)30203-0, PMID: 32437679 PMC7234781

[ref69] Sheline Y. I. Price J. L. Yan Z. Mintun M. A. (2010). Resting-state functional MRI in depression unmasks increased connectivity between networks via the dorsal nexus. Proc. Natl. Acad. Sci. USA 107, 11020–11025. doi: 10.1073/pnas.1000446107, PMID: 20534464 PMC2890754

[ref70] Sorg C. Riedl V. Muhlau M. Calhoun V. D. Eichele T. Laer L.. (2007). Selective changes of resting-state networks in individuals at risk for Alzheimer's disease. Proc. Natl. Acad. Sci. USA 104, 18760–18765. doi: 10.1073/pnas.0708803104, PMID: 18003904 PMC2141850

[ref71] Staffaroni A. M. Brown J. A. Casaletto K. B. Elahi F. M. Deng J. Neuhaus J.. (2018). The longitudinal trajectory of default mode network connectivity in healthy older adults varies as a function of age and is associated with changes in episodic memory and processing speed. J. Neurosci. 38, 2809–2817. doi: 10.1523/JNEUROSCI.3067-17.2018, PMID: 29440553 PMC5852659

[ref72] Szczepanski S. M. Knight R. T. (2014). Insights into human behavior from lesions to the prefrontal cortex. Neuron 83, 1002–1018. doi: 10.1016/j.neuron.2014.08.011, PMID: 25175878 PMC4156912

[ref73] Talairach J. Tournoux P. (1988). Co-planar stereotaxic atlas of the human brain: 3-dimensional proportional system: An approach to cerebral imaging, New York: Thieme Medical Publishers.

[ref74] Thaweethai T. Jolley S. E. Karlson E. W. Levitan E. B. Levy B. McComsey G. A.. (2023). Development of a definition of Postacute sequelae of SARS-CoV-2 infection. JAMA 330:1492. doi: 10.1001/jama.2023.15712 PMC10214179 37278994

[ref75] Varatharaj A. Thomas N. Ellul M. A. Davies N. W. S. Pollak T. A. Tenorio E. L.. (2020). Neurological and neuropsychiatric complications of COVID-19 in 153 patients: a UK-wide surveillance study. Lancet Psychiatry 7, 875–882. doi: 10.2139/ssrn.3601761, PMID: 32593341 PMC7316461

[ref76] Venkataramani V. Winkler F. (2022). Cognitive deficits in long Covid-19. N. Engl. J. Med. 387, 1813–1815. doi: 10.1056/NEJMcibr2210069, PMID: 36351274

[ref77] Winter D. Braw Y. (2023). Effects of diagnosis threat on cognitive complaints after COVID-19. Health Psychol. 42, 335–342. doi: 10.1037/hea0001286, PMID: 37141019

[ref78] Yuan H. Ding L. Zhu M. Zotev V. Phillips R. Bodurka J. (2016). Reconstructing large-scale brain resting-state networks from high-resolution EEG: spatial and temporal comparisons with fMRI. Brain Connect. 6, 122–135. doi: 10.1089/brain.2014.0336, PMID: 26414793

[ref79] Yuan H. Shou G. Gleghorn D. Ding L. Cha Y. H. (2017). Resting state functional connectivity signature of treatment effects of repetitive transcranial magnetic stimulation in mal de Debarquement syndrome. Brain Connect. 7, 617–626. doi: 10.1089/brain.2017.0514, PMID: 28967282 PMC5695731

[ref80] Yuan H. Young K. D. Phillips R. Zotev V. Misaki M. Bodurka J. (2014). Resting-state functional connectivity modulation and sustained changes after real-time functional magnetic resonance imaging neurofeedback training in depression. Brain Connect. 4, 690–701. doi: 10.1089/brain.2014.0262, PMID: 25329241 PMC4238245

[ref81] Zhang H. Chung T. W. Wong F. K. Hung I. F. Mak H. K. (2022). Changes in the Intranetwork and internetwork connectivity of the default mode network and olfactory network in patients with COVID-19 and olfactory dysfunction. Brain Sci. 12:511. doi: 10.3390/brainsci12040511, PMID: 35448042 PMC9029634

[ref82] Zhang F. Khan A. F. Ding L. Yuan H. (2023). Network organization of resting-state cerebral hemodynamics and their aliasing contributions measured by functional near-infrared spectroscopy. J. Neural Eng. 20:016012. doi: 10.1088/1741-2552/acaccb, PMID: 36535032 PMC9855663

[ref83] Zonneveld H. I. Pruim R. H. Bos D. Vrooman H. A. Muetzel R. L. Hofman A.. (2019). Patterns of functional connectivity in an aging population: the Rotterdam study. NeuroImage 189, 432–444. doi: 10.1016/j.neuroimage.2019.01.041, PMID: 30659958

